# Physical restraint within the prehospital Emergency Medical Care Environment: A scoping review

**DOI:** 10.1016/j.afjem.2023.03.006

**Published:** 2023-06-09

**Authors:** Jared MCDOWALL, Andrew William MAKKINK, Kelton JARMAN

**Affiliations:** 1 Netcare Education, Faculty of Emergency and Critical Care, Johannesburg, Gauteng, South Africa; 2 Department of Emergency Medical Care, University of Johannesburg, Doornfontein, Gauteng, South Africa

**Keywords:** Prehospital restraint, Aggressive patient, Aggressive patient risk mitigation

## Abstract

**Background:**

Psychomotor agitation and aggressive behaviour (AAB) have the potential to occur in any healthcare setting, including those in which Emergency Medical Services (EMS) operate. This scoping review aimed to examine the available literature on physical restraint of patients within the prehospital setting and to identify guidelines and their effectiveness, safety to patients and health care practitioners and strategies relating to physical restraint when used by EMS.

**Methods:**

We performed our scoping review using the methodological framework described by Arksey and O'Malley augmented by that of Sucharew and Macaluso. Several steps guided the review process: identification of the research question, eligibility criteria, information sources (CINAHL, Medline, Cochrane and Scopus), search, selection and data collection, ethical approval, collation, summarizing and reporting on the results.

**Results:**

The population of interest, in this scoping review was prehospital physically restrained patients, however, there was a reduced research focus on this population in comparison to the larger emergency department.

**Conclusion:**

The limitation of informed consent from incapacitated patients may relate to the lack of prospective real-world research from previous and future studies. Future research should focus on patient management, adverse events, practitioner risk, policy, and education within the prehospital setting.

**African relevance**

• The lack of information, pertaining to a validated tool/ guide for the management of AAB patients is concerning.
• The article provides some insight on proposed methodology.
• The study is an opportunity for further research to be done to assist all healthcare providers.

## Introduction

Psychomotor agitation can be defined as a feeling of excitement, and/or distress, which is associated with increased motor activity due to psychiatric/organic causes [1]. Aggressive behaviour in patients can be defined as threats (verbal and/or physical) towards healthcare providers, bystanders, and/or the patient themselves [1]. There is an association between increased psychomotor agitation and aggressive behaviour (AAB), which has the potential to occur in any healthcare environment, including the prehospital emergency medical services (EMS) environment [2].

Workplace violence (WPV) can be defined as an incident in which a staff member experiences AAB directed towards them by a patient, a family member, or a bystander in a work-related environment [3]. The incidence, and prevalence, of WPV against healthcare personnel (HCP) has constantly been increasing [4,5]. This has led to the development of several approaches to aid HCP in the management of AAB in patients, namely restrictive and/or coercive restraint approaches [6].

Within the South African prehospital setting, there is a lack of policy that relates to the restraint of patients and who assumes ultimate responsible for the restraint of these patients. Further to this, the Academy of Science of South Africa highlights that prehospital curricula have “insufficient content on emergency presentations of mental disorders in order to manage disorders safely and confidently” [7]. This is further compounded by the differences in training and capabilities of South African prehospital emergency care providers (PECP). The capabilities of South African PECP are regulated by the Health Professions Council of South Africa (HPCSA) and are dependent on the registration category of the healthcare practitioner [7]. These capability limitations translate into most prehospital HCP not having access to any chemical restraint agents. The result is that apart from coercive approaches, physical restraint is the mainstay of managing the AAB patient within the South African prehospital environment [8].

The lack of policy or guidelines extends to South African law enforcement where there appears to be a lack of clear guidelines or policy regarding AAB persons requiring medical attention.

The lack of clear roles or guidelines and the limited access to chemical restraint has identified a need to describe the available evidence related to physical restraint of AAB patients within the prehospital environment. This scoping review aimed to identify and examine physical restraint of patents within the prehospital context including clarification of definitions and concepts related to prehospital restraint, physical restraint techniques used to control AAB patients in the prehospital environment and to identify gaps for future research and/or systematic review.

## Materials and Methods

The format of the scoping review followed the methodological framework described by Arksey and O'Malley [9], as well as that of Sucharew and Maculuso [10]. and was reported on using the preferred PRISMA extension for scoping reviews (PRISMAScR) [11].

### Search strategy

A search strategy was developed and applied to the electronic database searches (July 2021), which included PubMed, CINAHL, Cochrane and SCOPUS. The following search terms were used: (1) ‘Physical restraint AND Paramedic’, (2) ‘Physical restraint AND Prehospital’, (3) ‘Physical restraint AND Emergency medical care’, (4) ‘Physical restraint AND Out-of-hospital’, (5) ‘Mechanical restraint AND Paramedic’, (6) ‘Mechanical restraint AND Prehospital’, (7) ‘Mechanical restraint AND Emergency medical care’, (8) ‘Mechanical restraint AND Out-of-hospital’, (9) ‘Coercive measures and Paramedic’, (10) ‘Coercive measures and Prehospital’, (11) ‘Coercive measures and Emergency medical care’, (12) ‘Coercive measure and Out-of-hospital’. The citations of the papers found were also reviewed for any other relevant articles. Language restrictions were applied.

### Study selection

Articles included in the review were required to meet specific criteria, related to AAB management within the prehospital environment. The primary inclusion criteria were determined by asking the following questions:

1. Was the source about physical restraint or coercion?
2. Was the source about prehospital care or EMS?
3. Was the source about emergency care of aggressive, agitated, or combative patients?
4. Was the source specifically focused on physical restraint or coercion?

### Data extraction and methodological evaluation

The format of the scoping review followed the methodological framework described by Arksey and O'Malley [9], as well as that of Sucharew and Maculuso [10]. The Preferred Reporting Items for Systematic reviews and Meta-Analyses extension for Scoping Reviews (PRISMA-ScR) flow diagram was followed to guide and record the search process (Figure) [11]. Each reviewer completed a descriptive narrative of eligible studies, and data were summarised and tabulated (Table 1).

**Figure:**
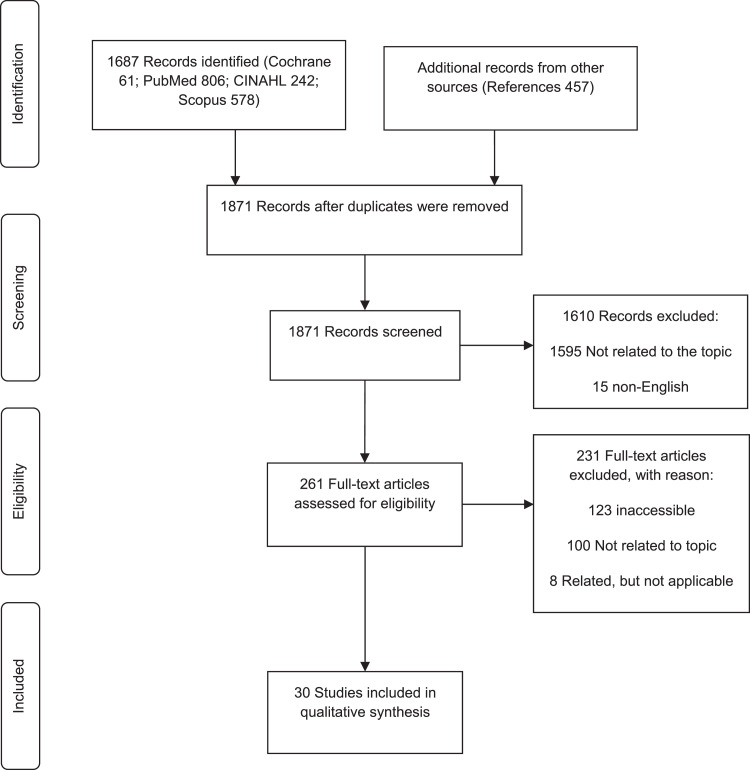

**Table 1:** Data charting form and result

Author/s, year, ^r^^e^^f^	Origin	Aim/s	Sample size	Methodology	Intervention type/ comparator	Author/s conclusion/s
Marks, 1992 [12]	USA^a^	Review physical restraint.	0	R^f^	PR^h^	No evidence to indicate benefit from physical restraint use.
O'Halloran *et al*., 1993 [13]	USA	Describes restraint asphyxia in excited delirium.	11	R	PR	Causes of SCA^j^ in hogtied patients outlined.
Tintinalli *et al*., 1993 [14]	USA	Identify violence frequency toward EMS and patient management.	32	R	AM^i^	50% of EMS providers had protocols in place. Risk identification and training is required.
Stratton *et al*., 1995 [15]	USA	Describe potential complications due to physical restraint/s.	2	R	PR	Hogtie poses asphyxia risk, mitigation processes are pivotal.
Abdon-Beckman, 1997 [16]	USA	Describe restraint by law enforcement.	0	R	PR	Reviews risks, adverse events, and legal considerations. Recommends risk minimisation review
Brauer *et al*., 1997 [17]	USA	Describe restraint use in aeromedical transport.	92	R	AM	65% of providers had a restraint protocol in place; common restraint devices included cloth and gauze.
Chan *et al*., 1997 [18]	USA	Evaluate the effect of hogtie restraint on respiratory function.	15	P^g^	PR	Clinically insignificant respiratory function changes from restraint positioning.
Chan *et al*., 1998 [19]	USA	Describe restraint physiology and hogtie restraint related death.	0	R	PR	No evidence to support hogties positioning effect on ventilation, other factors such as drug use must be investigated
Pollanen *et al*., 1998 [20]	CA^b^	Determine physical restraint frequency amongst excited delirium deaths.	21	R	PR	Hard physical restraint/s, excited delirium, male/s, & stimulant use are associated with SCA.
Hick *et al*., 1999 [21]	USA	Describe physical restraint related deaths & non-fatal encounters.	10	R	PR	Restraint struggle increases mortality; Hogtie not recommended.
Schmidt *et al*., 1999 [22]	USA	Evaluate restraint position on physiology after exercise.	18	P	PR	Clinically insignificant physiological changes from physical restraint.
Parkes, 2000 [23]	ENG^c^	Evaluate restraint position effect on recovery rate.	13	P	PR	Clinically insignificant physiological changes from physical restraint.
Park *et al*., 2001 [24]	USA	Describe agitated delirium, restraint, and death.	0	R	PR	Lateral/supine positioning preferred as least risk, continued struggle against restraint indicates need for chemical restraint due to risk, drug users are at risk for SCA.
Stratton *et al*., 2001 [25]	USA	Outline factors associated with death related restraint.	282*	R	PR	Risk factors for death in restraint- Obesity, Stimulant drug use, evident forceful struggle, RDS^k^, & chronic disease.
Brice *et al*., 2002 [26]	USA	Describe violence against EMS and the use of restraint.	0	R	PR	Indications for restraint are outlined; hogtie & hard restraints not recommended for routine use.
Chan *et al*., 2004 [27]	USA	Evaluate respiratory effect of prone restraint.	10	P	PR	Clinically insignificant respiratory changes from restraint.
Cheney *et al*., 2006 [28]	USA	Identify factors of assault on EMS by restrained patients.	77⁎⁎	R	AM	Identifies risk factors of assault to EMS^l^ personnel by restrained patients.
Kim *et al*., 2007 [29]	USA	Details assessment & management of agitated children.	0	R	AM	Physical restraint techniques, EMS training and protocol adherence
Macias *et al*., 2007 [30]	USA	Develop a scale to determine likelihood providers use restraint.	22	P	PR	Tool designed to compare providers likelihood to emergency mechanical restrain, Danger to self and severe agitation indicated to have a 0.87 physical restraint incident.
Michalewicz *et al*., 2007 [31]	USA	Investigate restraint and the associated effects on ventilation and metabolism.	30	P	PR	Clinically insignificant physiological changes from physical restraint.
Campbell *et al*., 2008 [32]	USA	Evaluate a restraint training module for paramedic students.	34	P	PR	Training will affect restraint use by EMS.
Vilke *et al*., 2011 [33]	USA	Investigate effect of restraint chair on respiratory function.	10	P	PR	Maximal voluntary ventilation reduced; no significant effects seen.
Weiss *et al*., 2012 [34]	USA	Determine the efficacy of restraint in agitation management.	618	P	AM	Chemical restraint efficacy outweighs physical restraint.
Savaser *et al*., 2013 [35]	USA	Evaluate restraint position and additional weight effects on cardiac function.	25	P	PR	Clinically insignificant cardiac changes from physical restraint use.
Sloane *et al*., 2014 [36]	USA	Evaluate restraint position in obesity after exercise.	10	P	PR	Clinically insignificant physiological changes from physical restraint.
Strote *et al*., 2014 [37]	USA	Evaluate individuals identified by law enforcement to be exhibiting excited delirium.	43	R	AM	Identification of excited delirium did not show increased complication/s.
Nambiar *et al*., 2020 [38]	ASNZ^d^	Describe the care and assessment of the agitated patient.	0	R	AM	Differences in assessment and management of agitation.
Dijkhuizen *et al*., 2020 [39]	NL^e^	Identify cause of death in law enforcement-initiated restraint.	186	R	PR	Identifies cause of SCA as multifactorial- Obesity, Drug use, male gender, naked, 30-40 years higher incidence in research group
Neilson *et al*., 2020 [40]	CA	Review literature & policies for mental health transfers.	0	R	PR	Psychiatric transfers and restraint are understudied; outlined the associated harm & a decreased frequency in the utilisation of physical restraint/s.
Sloane *et al*., 2020 [41]	USA	Investigate physiological effects of stress whilst retrained.	25	P	PR	Co-peptin levels were higher in stressed, No significant effects found

a United States of America

b Canada

c England

d Australia and New Zealand

e Netherlands

f Retrospective

g Prospective

h Physical restraint

i Agitation management

j Sudden cardiac arrest

k Respiratory distress syndrome

l Emergency medical services

⁎ - Average of 47 restraint procedures over 6 years

⁎⁎ - 77 of 271 restrained patients during a one-year study

## Results

### Search

The database search identified a total of 1687 articles: PubMed (806), Scopus (578), CINAHL (242) and Cochrane (61). The reference lists of the articles were incorporated as additional articles (457). There were 1871 total articles screened, after which 273 duplicates were identified and removed. Screening included title and abstract assessment. The screening process excluded 1595 unrelated and 15 non-English articles. There were 261 articles that were assessed for eligibility. There were 231 ineligible articles: 123 were inaccessible, 100 were unrelated and eight were related, but not applicable. The 123 articles that were inaccessible, were not available online, nor could the University librarian access the articles.

### Dataset range

There were 23 articles published more than 10 years ago [12], [13], [14], [15], [16], [17], [18], [19], [20], [21], [22], [23], [24], [25], [26], [27], [28], [29], [30], [31], [32], [33], [34], three in the last 10 years [35], [36], [37], and our articles in the last five years [38], [39], [40], [41].

### Country of origin

Most of the articles were concentrated in North America, with the United States of America contributing 25 articles [[12], [13], [14], [15], [16], [17], [18], [19],^21^,^22^,[24], [25], [26], [27], [28], [29], [30], [31], [32], [33], [34], [35], [36], [37],^41^], and Canada two [20,40]. There was one article from England [23] and one from the Netherlands [39], as well as one combined article from New Zealand and Australia [38].

### Study Aims

The articles revealed similar characteristics in terms of the aim/s and/or purpose/s. The review or description of physical restraint concepts was focused on in 10 articles [12,13,15,[19], [20], [21],^24^,^25^,^29^,^38^]. There were nine articles that investigated the physiological harm and/or disturbance caused by physical restraint [18,22,23,27,31,33,35,36,41]. There were six articles that described physical restraint and violence against prehospital providers [14,16,17,26,28,37], further, two articles described physical restraint application/use by law enforcement personnel [20,39].

There were two articles that described the application of Video Assessment of Propensity to Use Emergency Restraints Scale (VAPERS) [30,32], and one article compared the efficacy of physical restraint and chemical restraint [34].

### Methodology

The reviewed articles were divided into 18 retrospective [[12], [13], [14], [15], [16], [17],[19], [20], [21],[24], [25], [26],^28^,[37], [38], [39]] and 12 prospective articles [18,22,23,27,[30], [31], [32], [33], [34], [35], [36],^41^]. Recurrent methodologies included eight review articles, eight randomized experimental control articles, four cross-sectional articles and three case studies.

### Intervention type and/or comparator

The revised intervention type themes found were agitation management in seven articles [14,17,28,29,34,37,38], and physical restraint in 23 articles [12,13,15,16,[18], [19], [20], [21], [22], [23], [24], [25], [26], [27],[30], [31], [32], [33],^35^,^36^,[39], [40], [41]].

### Key themes and authors’ conclusion/s

Key concepts relating to the articles were identified and are summarized below:

#### Agitated patient assessment and management

There were 12 articles that reported on the assessment and management of agitation [12,14,17,21,24,26,[28], [29], [30],^32^,^34^,^38^]. These studies described assessment tools to rate agitation and outlined the indications for intervention/s [12,14,17,21,24,26,[28], [29], [30],^32^,^34^,^38^]. Management focused on restraint; verbal, physical and/or chemical restraint [21,24,26,29]. Physical restraint was divided into soft and hard type techniques. Hard type techniques included prone restraint, hogtie/s, and the use of bodyweight force applied to the posterior thorax or neck [19,27,31,35,36,41]. Articles indicated that law enforcement may initiate or maintain hard type techniques to AAB patients, an environment not dissimilar to that in which EMS operate [37,39,41]. Soft type restraint/s were associated with EMS intervention/s [17,28,29,34,38]. Soft-type restraint was described in four articles and comprised of four-point padded restraints in supine, lateral or seated body positioning [16,21,24,29].

Management principles focused on preventing harm, facilitating the identification and treatment of AAB aetiology [17,24,26,29,30,32]. The efficacy of physical restraint, in controlling agitation, has been questioned, and it was concluded that there was no clear evidence of patient benefit in any situation outside of an emergency [12,34]. Physical restraint may not adequately control severe agitation, and resistance to restraints may occur in up to 20% of restrained patients [34]. Progression to chemical restraint has been recommended as an escalation of care [21,24,26,29,34].

#### Restraint position and related physiology

There were 18 articles that reported on patient body position, whilst restrained, and the resulting effect on physiology [13,15,16,18,19,[21], [22], [23], [24], [25], [26], [27],^31^,^33^,^35^,^36^,^39^,^41^]. Hogtie or prone-maximal-restraint-positioning, with or without weight force to the back, or with submaximal exercise was investigated. The articles described that although small changes in physiological parameters were measured, the changes were clinically insignificant [13,15,16,18,19,[21], [22], [23], [24], [25], [26], [27],^31^,^33^,^35^,^36^,^39^,^41^].

#### Adverse events and mitigation

There were 20 articles that reported on physical restraint adverse events and mitigation recommendations [12,13,[15], [16], [17],[19], [20], [21], [22], [23], [24], [25], [26], [27], [28], [29],^34^,^37^,^38^,^40^]. The adverse events ranged from negative psychological effects to minor and severe physical injury (worst being fatal outcome/s) [12,13,[15], [16], [17],[19], [20], [21], [22], [23], [24], [25], [26], [27], [28], [29],^34^,^37^,^38^,^40^]. Patients reported a feeling of stigmatization, criminalization, and fear when they were physically restrained [12,40]. Patients further described the experience to be traumatic and resulted in decreased trust towards prehospital personnel [12]. Physical injury was identified as trauma inflicted to patients; during the application of restraint/s or whilst struggling in restraint/s [39]. Trauma such as minor contusions, abrasions, fractures, and brain injuries were reported [28,39].

Restraint asphyxia, excited delirium and recreational drug toxicity were identified as causes of death during physical restraint [13,15,[18], [19], [20],^25^,^39^]. Further, Obesity, hyperthermia, metabolic acidosis, drug-use, chronic disease, and respiratory distress were associated with sudden cardiac arrest [18,20,21,25,37,39].

To reduce the risk of physical injury, due to hard type restraint, it was recommended that gauze and soft padded restraint materials be added [12,28,29]. Lateral or supine positioning was preferred due to interference with vital sign/s monitoring (in prone position), and a high association of death related adverse events with face-down restraint positioning [16,23,24,26,29]. Ongoing severe agitation and struggling, despite physical restraint application, was identified as an immediate emergency and escalation to chemical restraint was recommended [24,26,29,34].

#### Practitioner risk

There were eight articles that reported on physical restraint and risks associated among prehospital personnel and that assault and violence against HCP was a prominent risk [14,17,26,28,29,34,38,40]. Females, patients accessed between midnight and 6AM, as well as patients injured under supervision within the prehospital setting were more likely to be aggressive and assault HCP [28]. The perceived need for chemical restraint administration by HCP was linked to higher incidences of assault [28,34]. The incidence of assault was reported in 28% of patients that were restrained [34].

Risks such as bites, blood in the eye and needle-stick injuries, resulted in biohazardous or infectious disease risk [26,29]. The use of personal protective equipment like helmets, face shields and body armor were documented to protect providers [26,29].

#### Education and Policy

There were nine articles that reported on education and policy pertaining to restraint [14,16,17,26,29,30,32,38,40]. It was found that the incidence of protocol-use when restraining patients ranged between 50 to 65% [14,17,26]. Articles recommended that EMS providers institute policies to guide their staff in situations requiring the restraint of a patient [14,16,17,26,29,30,32,38,40]. Policies were unstandardized with variation in agitation assessment tools, specific protocols, and reporting [14,16,17,26,29,30,32,38,40]. Reports and surveys indicated that few providers had formal training on AAB management, identification, and related concepts of treatment [14,17,26]. The education of physical restraint concepts was recommended, with an article further evaluating the effect of training on the propensity to restrain in prehospital students [17,26,32,38].

The VAPERS has been used to demonstrate how low-restraint-propensity providers may increase their propensity to provide restraint in certain situations and, that training providers that have a high-restraint-propensity may decrease their propensity to restrain [32]. The VAPERS had been developed to assess propensity to apply emergency restraint by providers in all settings and demonstrated that severe agitation and danger to self were the primary driving factors in making the decision to restrain a patient with 87% of providers restraining such patients [30].

#### Legal responsibility

A total of six articles reported legal responsibility and associated aspects pertaining to physical restraint [12,14,16,17,29,40]. The patients’ rights and adherence to local laws and protocols was commonly highlighted, ethical concerns and patient autonomy were also considered [12,14,16,17,29,40]. The providers expressed concern in situations whereby inadequate levels of restraint, resulted in preventable harm and further litigation [40]. In summary, legal aspects were associated with the decision to restrain a patient and the level of appropriateness with regards to the type of restraint.

## Discussion

This scoping review provides an overview of currently available literature relating to physical restraint within the prehospital emergency medical care context. Based on the results of this review we have identified several themes as well as certain gaps in the literature. The review highlights key assessment and management techniques related to the AAB patient, restraint position and physiological consequences of restraint, adverse events and mitigation strategies, risk to practitioners, education and policy related to physical restraint and, legal responsibilities pertaining to physical restraint. These themes were reviewed, and we have suggested some recommendations related to physical restraint of patients as well as identifying knowledge gaps and areas for future research.

### Assessment and management of the agitated patient

Several agitation assessment tools assist practitioners in decision-making have been described in the literature [32,34,38]. The Agitated Behaviour Scale has been validated for emergency department use and its use prehospitally has been described as having a good fit. Unlike prehospital screening tools for common conditions such as suspected stroke, there are no validated screening tools for the prehospital AAB patient. Prehospital validation for screening the AAB has been recommended for future studies [34]. The use of the Sedation Assessment Tool (SAT), the Mental Health Triage Tool and Glasgow Coma Scale have been described in New Zealand and Australia [38]. The use of agitation assessment tools facilitates baseline and progressive assessment when incorporated as part of a dedicated restraint policy [32,34,38]. Agitation assessment tools have been used in settings other than the prehospital, but their increased use may allow for more consistent definition of agitation severity and better communication relating to agitation between healthcare providers [30,32,34].

It is important to assess and explore the causes of agitation which may be varied and often result from conditions that cause an altered level of consciousness [12,26,29]. The causes may include, but are not necessarily limited to, infection, alcohol intoxication, drug overdose or poisoning, seizures, psychosis, electrolyte and metabolic abnormalities or any other condition that has the potential to cause an altered level of consciousness [12,26,29,42].

Treating the root cause may provide mechanisms to directly reduce agitation but in the prehospital environment, narrowing the differential is difficult, making treating the root and reducing agitation difficult [12,26,29]. One of the challenges related to assessment and management of the agitated patient is that there is a lack of standardization to guide practitioners in best practice principles [14,16,17,26,29,38,40]. This is compounded by sources such as Marks (1992) who concluded that restraints were overused in medicine and that there was limited (if any) evidence to support their efficacy [12]. Contextually, this article is old and did not include prehospital data but highlights the incongruences that exist related to patient restraint.

It is unfortunate that the restraint of AAB patients remains a treatment of necessity. The predominant indication identified for restraining a patient related to risks to the immediate safety of person/s and/or property [17,24,26,30]. Restraining the AAB patient allows for the provision of an environment in which assessment, life-saving interventions and transportation to definitive care can be carried out in relative safety [17,24,26,30]. Managing the AAB patient often follows a path of escalation commencing with verbal de-escalation techniques and escalating to physical restraint and/or chemical restraint in severe cases [21,24,26,29].

Physical restraint has been reported as having poor efficacy in adequately managing agitation [21,26,29,34]. In cases where severe or ongoing agitation is encountered, patients require escalation from physical to chemical restraint [21,24,26,29,34].

It is interesting to note that ineffective management of agitation in physically restrained patients has been associated with a perceived need for chemical restraint in 77% of patients. In addition, the authors noted that 44% of patients exhibited sustained agitation and 29% obstruction of assessment. Chemical restraint has been associated with the better prevention of obstructive and detrimental patient behaviours that are difficult to manage using physical restraint techniques alone [24,26].

The escalation to chemical restraint improves movement restriction for assessment and treatment and has been used in critical and life-threatening populations [24,26]. Once the decision has been made to physically restrain a patient, positioning and related physiological consequences require evaluation.

### Restraint positioning and related physiological responses

The primary aim of restraint position is to minimise the movement of a patient into a predetermined position, whilst taking appropriate cautionary steps to prevent harm. This aim is achieved by providers gaining control of a patient's body using physical means [12,29]. The very nature of this process often means that additional manpower is required, and some EMS policies suggest waiting for law enforcement prior to initiating physical restraint procedures [26,28,38]. Once the patient has been manually restrained, restraint devices are placed onto the limbs which are physically held by personnel until having been secured in the relevant position using the most appropriate means [12,29]. The process of restraining a patient usually involves four to six people and a four- or six-point restraint systems being recommended, often with the addition of a thoracic and pelvic strap [16,21,24,29,43]. Auxiliary adjuncts used in the restraint process included restraint chairs, electrical tasers and pepper spray, although these were used by law enforcement and not EMS personnel [20,33,37,41]. Recommended positions for restraint included allowing the patient to remain seated as well as placing them in the supine or lateral position on a stretcher as well as the hog-tie restraint position [16,18,19,21,24,29].

The maximum prone restraint or hog-tie position is one of the more restrictive positions and involves the patient being held prone whilst the upper and lower limbs are pulled together around the posterior midsection with metal or leather handcuffs, tape, Velcro, or other adjuncts fitted to maintain the position [12,14,17,28,29]. This position is primarily used by law enforcement personnel for in-custody positioning and unrelenting confinement and control [37,39,41]. Hard type restraint materials and methods are not recommended for prehospital use [17,26,28,29,34,38].

The physiological effects of positional restraint have been described in several studies, but there has been no conclusive or direct link between physiological changes and patient death [18,19,[22], [23], [24],^26^,^27^,^31^,^33^,^35^,^36^,^41^]. In addition, studies evaluating the simulation of a struggling patient, the added weight force applied to the surface upon which the patient was lying and alternating restraint positions were also evaluated [22,27,35,36]. Although a decrease in respiratory volume was described, this was of low clinical significance and that patient death was likely due to other confounding causes [18,19,[22], [23], [24],^26^,^27^,^31^,^33^,^36^,^41^]. One of the limitations of many of these studies was their small sample size which limits their generalisability.

### Patient injury due to restraint

Several sources question the benefit of physical restraint due to the potential for adverse events in a population that is particularly vulnerable [12,40]. Dijkhuizen *et al* (2020) described that only 2.6% of restrained patients who demised in custody did not have evidence of physical trauma [39]. Injury to patients because of restraint can be divided into three major categories: minor, moderate, or severe. These categories are linked to the severity of the injury and its potential consequences.

#### Minor injury

Minor injuries most occurred during the process of restraint or due to patients’ continued resistance to the securing device(s). These injuries have been described as superficial and include contusions, abrasions, and lacerations [12,28,34]. The incidence of minor adverse events has been estimated to range between less than two percent and thirty-one percent in patients who were restrained [28,34,39]. In cases where total control may be required from physical restraint, there is a dependence of the patient on providers for normal life processes. If this is poorly managed, avoidable adverse effects such as urinary or faecal incontinence may occur which may be considered minor physical and psychological injury [12].

#### Moderate injury

Moderate injury includes physical and psychological injury: fractured bones, dislocation/s and other non-life-threatening injuries such as compression neuropathy that are considered with more serious than minor injuries [39,40]. Dijkhuizen *et al* (2020) reported that in autopsy reports of restraint patients, 29% exhibited evidence of moderate trauma [39].

Psychologically patients who experienced restraint have described feelings of fear, dehumanisation, trauma, punishment, and have expressed perceptions of stigmatisation, as well as loss of therapeutic trust with the practitioner when placed into physical restraints [12,40]. Mental health users are more likely to experience emotional trauma because of restraint than are non-MHUs and there is a risk of exacerbating post-traumatic stress.

Both patients and their families are exposed to these psychological risks that have the potential for long-term psychological injury [40]. The use of verbal de-escalation has the potential to mitigate the need for restraint and in doing so encourage better therapeutic relationships and decrease the incidence of adverse event [29,40].

#### Severe injury

Physical restraint and death have been the topic of several news reports and there are numerous studies exploring this phenomenon [13,15,16,21,23,24,39,44]. Severe and life-threatening injuries include closed traumatic brain injury and liver laceration [39]. Populations most at risk for cardiac arrest during restraint include the male obese patient, active psychosis, stimulant drug use and underlying medical conditions [13,20,24,39,44]. Dijkhuizen *et al* (2020) reported that in autopsy reports of restraint patients who had demised, 7.9% of the injuries documented were life-threatening [39]. Importantly, Stratton *et al* (1995, 2001) identified the progression of a combative patient resisting against the restraint to a flaccid state as a hallmark trait of a fatal outcome [15,44].

The positioning of restrained patients has gained attention due to multiple reports of restraint-associated death [13,15,16,21,24,39,44]. Prone or hogtie positioning have been commonly linked to death of a restrained patient [13,15,16,[18], [19], [20],^24^,^44^]. There were 77 accumulative reports of death during restraint in the prone position with the hands and ankles bound behind the back and these were attributed to an increased susceptibility to asphyxia due to active psychotic states or drug-induced delirium and the associated increased physiological demands [13,15,16,21,24,39,44].

Death is the most severe adverse event and Stratton *et al* retrospectively described 18 witnessed EMS cardiac arrests after struggle and physical restraint over a six-year period [44]. Mortality after cardiac arrest during restraint was found to be 100% by both Pollanen *et al* (1998) and Stratton *et al* (2001) despite resuscitation efforts, highlighting the importance of recognition of risk factors and prevention of cardiac arrest [20,44]. It is important to continually monitor restrained patients for early detection of physiological abnormalities and in the event of cardiac arrest it is critical that prehospital providers have adequate access to the patient such that they can perform cardiopulmonary resuscitation [16,29]. Reducing the use of hard-type restraint techniques and the promotion of lateral or supine positioning may reduce the risk of adverse events [16,23,24,26].

### Identification of adverse events in the restrained patient

Critical to mitigation strategies is an understanding of the related compounding factors. The use of stimulant drugs, such as cocaine, has been linked to a high correlation with sudden cardiac arrest in patients who have been physically restrained [24,39,44]. In addition, recreational drug toxicity has been implicated amongst physical restraint-related deaths [13,15,20,21,39,44]. The role of hyperthermia in the demise of physically restrained patients, with or without stimulant use, remains unclear, but excited delirious states (ExDS) that are characterised by agitated delirium, hyperthermia and respiratory arrest have been purported to play a significant role in restraint-related deaths [16]. Interestingly, Strote *et al* (2014) found that patients exhibiting signs of excited delirium did not necessarily exhibit a higher complication rate but acknowledged that it was possible that the officers in their study had misidentified subjects experiencing ExDS [37]. Sudden cardiac arrest during restraint has been correlated with metabolic acidosis and excited delirium but there has been no link made with these as causative agents [21,37]. The application of physical restraints may increase agitation and patients who struggle against the restraints may present with metabolic abnormalities and multiple system complications. Additionally, continuous struggle against restraints has been identified as potentially life-threatening and requires appropriate intervention by the healthcare practitioner [12,24,26,29,34]. Obesity has also been identified as a potential risk factor in physical restraint due to there being a moderate presence in fatal outcomes of physically restrained individuals, further highlighting the need for close and accurate monitoring [39,44].

Restraint asphyxia has the potential to occur due to either the initial restraint process or shortly after prone restraint has been achieved [15,16,18,19,44]. Reasons postulated for this include restrictive measures applied to the neck or thoracic region of the patient, as evidenced by reports from autopsies of physical restraint associated sudden cardiac arrest. These autopsy reports documented evidence of contusions around the neck and conjunctival petechial haemorrhage [18,20,44].

The importance of continual patient respiratory monitoring must be emphasised as a potential mitigation strategy.

### Practitioner risk

The practitioner should be cognisant that the agitated patient is likely to resist restraint at all costs and that there is a real risk of physical assault injuries such as biting, punching, and kicking during the primary restraint process [29]. It is important for prehospital practitioners to consider the confined space presented by a moving or stationary ambulance when physically restraining the patient and the effect that this may have on the manpower required during the restraint [14]. It is possible that should the decision to restrain a patient not be made prior to loading the patient into the ambulance, that it may not be possible once the agitated patient is inside [14,45].

There is a real risk to prehospital providers for injury on duty during restraint of the AAB patient with the risk as high as 61-67% [14,17,26]. Even in the restrained patient, the risk is as high as 27%, 4% of whom incurred physical injury, of which 1% required medical attention [32]. Given that agitated patients who require restraint have been known to have weapons such as knives in their possession, it is recommended that prehospital providers be exposed to violence management and situational awareness training [14]. It is often prudent for the prehospital provider to standby until law enforcement arrives and implement scene safety strategies. It is also recommended that personal protective equipment such as body armour and helmets be worn when restraining agitated patients [14,26,38]. Adequately analysing assault on duty reports can provide important insights related to risk identification and mitigation [38]. Variables linked to a high risk for assault included time of day (specifically between midnight and 06:00), the perceived or actual need for chemical restraint, patients of female gender, patients who had already exhibited violence, patients who had been injured whilst under supervision and patients already in custody [28,34]. These factors increase the risk of physical, emotional and biohazardous events and outline the need for violence management and avoidance training.

### Education and policy considerations

Education and appropriate policies regarding practitioners and physical restraint of patients are pivotal to mitigating risks and avoiding adverse events to both the prehospital provider and the patient [14,17,26,29,38,40]. Education programs focussing on terminology, causative pathologies, AAB, and mental capacity assessment have been shown to be beneficial in the management of the agitated patient. It must be noted that recent evidence appears to be lacking related to restraint programs in place and their effect/s [32]. Despite the benefits of structured policy, and recommendations that these are implemented, reports indicate that between 35% and 50% of EMS agencies do not have such policies in place [14,17,26]. Recommendations indicate that policies related to restraint should assist with assessment, diagnosis and management of agitated patients and should also cater for unique patient presentations [14,17,29,38,40]. Recommendations also exist for policies to guide clinical judgement and documentation along with accurate descriptions to facilitate review of cases [14,17,26,38].

### Legal responsibility

Marks (1992) highlighted the importance of a patient's freedom of movement and the responsibility to carefully consider the need for and to prove the necessity of restricting patients’ movements [12]. This is critical because a restraint intervention is typically against patients’ wishes and may be considered a violation of a patient's right to refuse treatment, resulting in the potential for litigation [12,14,29]. Death of a patient whilst under restraint raises several ethical and legal questions that are often linked to the reasons and decision-making process that ultimately led to the restraint [13,15,16,21,23,39,44].

Equally important as the decision to restrain, is the decision not to restrain, where omitting to restrain a patient may result in significant adverse events; self-harm including suicide or harm to the practitioner [45].

Policy formulation and implementation should include appropriate definitions and highlight liability concerns related to contextual and circumstantial due care and coupled with the appropriate indications for restraint. This should include involvement of local law enforcement stakeholders, consideration for the implications of local legislation and clear scope of responsibilities for all stakeholders [1,12,14,16,29,40].

## Limitations

The search limitations are a limitation to this scoping review and the exclusion of chemical restraint may have limited the generalisability of the results. We did not find any local prehospital studies which potentially limits the local relevance of the data sourced during the review. Most data were sourced from law enforcement agencies and although the environment is similar, this further limits the generalisability of the results. The small sample sizes of the sources included in the review may limit the applicability of the data to the clinical context and the non-experimental nature of some of the sources may further limit the conclusiveness of the sources.

## Recommendations

We identified several gaps in the literature and use the outcomes of this review to make recommendations based on some of the conclusions discussed in the included literature. There was limited epidemiological data pertaining to physical restraint in the prehospital setting and research that was available had a narrow distribution, creating a wide knowledge gap. There were significant gaps and differences in sub-contexts such as the prehospital validation of assessment tools used in other domains and between rural and urban, developed and developing countries, as well as primary response and interfacility transfer. We recommend that further research be conducted in assessing, evaluating and where relevant validating assessment tools that may have relevance within the prehospital environment.

We further recommend that policy review and updates be undertaken to determine areas requiring improvement and appropriate strategies to facilitate education and training programmes. There is clarity needed on roles and responsibilities in managing the AAB patient as well as what escalation and restraint strategies should be advocated.

There is a need for future research to focus on the identification, assessment and evaluation of appropriate patient assessment and diagnostic tools for the agitated patient, the actual and potential risk for adverse event to both patient and practitioner as well as appropriate escalation strategies. These should be concomitantly developed with relevant education and training interventions and policy development.

## Dissemination of Results

Results from this research were shared with staff members at the University of Johannesburg (research report and presentation). The results will also be published in a reputable international medical journal.

## Declaration of Competing Interest

The authors hereby certify that this submission is not under publication consideration elsewhere and is free from any conflict of interest.
